# The Effect of Liposomal Curcumin as an Anti-Inflammatory Strategy on Lipopolysaccharide e from *Porphyromonas gingivalis* Treated Endothelial Committed Neural Crest Derived Stem Cells: Morphological and Molecular Mechanisms

**DOI:** 10.3390/ijms22147534

**Published:** 2021-07-14

**Authors:** Francesca Diomede, Luigia Fonticoli, Simone Guarnieri, Ylenia Della Rocca, Thangavelu Soundara Rajan, Antonella Fontana, Oriana Trubiani, Guya Diletta Marconi, Jacopo Pizzicannella

**Affiliations:** 1 Department of Innovative Technologies in Medicine & Dentistry, University “G. d’Annunzio” Chieti-Pescara, 66100 Chieti, Italy; luigia.fonticoli@unich.it (L.F.); ylenia.dellarocca@unich.it (Y.D.R.); oriana.trubiani@unich.it (O.T.); 2 Department of Neuroscience, Imaging and Clinical Sciences, Center for Advanced Studies and Technology (CAST), University “G. d’Annunzio” Chieti-Pescara, 66100 Chieti, Italy; simone.guarnieri@unich.it; 3 Department of Biotechnology, Karpagam Academy of Higher Education, Coimbatore 641021, India; tsrajanpillai@gmail.com; 4 Department of Pharmacy, University “G. d’Annunzio” Chieti-Pescara, 66100 Chieti, Italy; Antonella.fontana@unich.it; 5 Department of Medical, Oral and Biotechnological Sciences, University “G. d’Annunzio” Chieti-Pescara, 66100 Chieti, Italy; guya.marconi@unich.it; 6 “Ss. Annunziata” Hospital, ASL 02 Lanciano-Vasto-Chieti, 66100 Chieti, Italy; jacopo.pizzicannella@unich.it

**Keywords:** curcumin, liposome, human periodontal ligament stem cells, endothelial-differentiation, *Porphyromonas gingivalis*, reactive oxygen species, inflammation, cardiovascular disease

## Abstract

Curcumin, a yellow polyphenol extracted from the turmeric root is used as a diet supplement. It exhibits anti-inflammatory, antioxidant, and antitumor properties by modulating different intracellular mechanisms. Due to their low solubility in water, the curcumin molecules must be encapsulated into liposomes to improve the bioavailability and biomedical potential. For the periodontal tissue and systemic health, it is essential to regulate the local inflammatory response. In this study, the possible beneficial effect of liposomes loaded with curcumin (CurLIP) in neural crest-derived human periodontal ligament stem cells (hPDLSCs) and in endothelial-differentiated hPDLSCs (e-hPDLSCs) induced with an inflammatory stimulus (lipopolysaccharide obtained from *Porphyromonas gingivalis*, LPS-G) was evaluated. The CurLIP formulation exhibited a significant anti-inflammatory effect by the downregulation of Toll-like receptor-4 (TLR4)/Myeloid differentiation primary response 88 (MyD88)/nuclear factor kappa light chain enhancer of activated B cells (NFkB)/NLR Family Pyrin Domain Containing 3 (NLRP3)/Caspase-1/Interleukin (IL)-1β inflammation cascade and reactive oxygen species (ROS) formation. Moreover, the exposure to LPS-G caused significant alterations in the expression of epigenetic modifiers, such as DNA Methyltransferase 1 (DNMT1) and P300, while the CurLIP treatment showed physiological expression. Overall, our in vitro study provides novel mechanistic insights into the intracellular pathway exert by CurLIP in the regulation of inflammation and epigenetic modifications.

## 1. Introduction

Regenerative medicine represents the forefront of health sciences, and it is based on the use of stem cells, including adult mesenchymal stem cells (MSCs), in order to produce tissue regeneration.

MSCs are multipotent cells that arise from the mesoderm that avoid the ethical concerns related to the use of stem cells. They are characterized by two main properties: ability to differentiate into different lineages, and self-renewal [1]. As reported previously, MSCs, when exposed to specific in vitro conditions, are able to differentiate towards endodermic, mesodermal and ectodermal lines; consequently, they are able to differentiate into bone cells, adipose cells, chondrocytes, muscle cells, liver cells, islet cells, and neurons. Since hematopoietic cells are similar to MSCs, hematopoietic markers, such as cluster of differentiation (CD) 14, CD34, CD45, and Human Leukocyte Antigens (HLA)-DR, are used to distinguish MSCs from hematopoietic cells [2,3,4,5]. There are several fetal sources of MSCs, such as amniotic fluid, umbilical cord, amniotic membranes, or placenta, but the interesting aspect is the chance to obtain MSCs in adult tissues including bone marrow and adipose tissues. In recent years, alternative sources of adult MSCs have been identified in the oral cavity tissues, which include dental pulp, apical papilla, dental follicle, gingiva, and periodontal ligament [6,7]. The MSCs isolated from the oral cavity niche showed the ability to adhere to plastic culture dishes, and they are able to expand significantly through consecutive in vitro passages without any modifications in the stemness profile [8,9]. Moreover, MSCs possess immunoregulatory properties and are capable of influencing both adaptive and innate immune responses by actively interacting with the components of the immune system and showing anti-inflammatory effects [10,11].

In particular, human periodontal ligament stem cells (hPDLSCs) have shown the ability to differentiate into mesengenic lineages and protect against infectious diseases by demonstrating immunomodulatory properties [12,13]. Indeed, they play an active role in the immune response thanks to the interaction with immunity cells, avoiding the improper activation of T lymphocytes, modulating the immune response during healing processes [14,15]. Current research is evaluating the response of MSCs to inflammatory events and the use of natural antioxidant agents.

Periodontitis is a chronic inflammation sustained by various types of gram-negative bacteria that lead to destruction of teeth supporting tissue [16]. Systemic diseases are related to a chronic inflammatory process, and the periodontal disease could represent a possible risk factor for cardiovascular disease (CVD); indeed, the bacteria placed in the periodontal pockets can be disseminated in the bloodstream to reach the endocardium tissue [17]. The biological mechanism underlying the relationship between oral health and CVD still needs to be elucidated [18].

Curcumin (Cur), a natural bioactive polyphenolic compound, is isolated from the rhizome of Curcuma longa Linn and largely insoluble in water. Several studies have reported that Cur has many pharmacological activities: antioxidant, anti-inflammatory and anti-cancer [19]. The antioxidant action of curcumin is related to its effect on reactive species: it eliminates superoxide anion (O−), peroxynitrite (NOO), nitric oxide (NO), peroxyl radicals (ROO), and hydroxyl radicals (OH−), causing a consequent upregulation of antioxidant proteins. In particular, the phenolic groups of Cur allow it to react with reactive species and could probably be one of the mechanisms by which cells are protected from oxidative damage following the administration of Cur. It may indirectly induce the expression of antioxidant proteins such as superoxide dismutase (SOD), catalase (CAT), glutathione peroxidase (GPx), glutathione reductase (GR), glutathione-S-transferase (GST) and g-glutamyl cysteine ligase (gGCL) [20]. Several in vitro and in vivo studies have shown that Cur gives promising results in the treatment of wound healing and inflammatory diseases.

Toll-like receptors (TLRs) play a key role in triggering the innate immune response and inflammation. Previous studies revealed that TLR is one of the major molecular targets of Cur, where it exhibited an inhibitory impact [21]. The myeloid differentiation factor-88 adaptor protein (MyD88) modulates most TLRs signaling as well as Toll/Interleukin receptor domain signaling through the interleukin (IL)-1 and IL-18 receptors. TLRs control the innate immunity via activation of the nuclear factor kappa-light-chain-enhancer of activated B cells (NFkB) and mitogen-activated protein kinases (MAPK) pathways and consequent generation of inflammatory cytokines and chemokines [22].

It has been shown that Cur is able to inhibit some pro-inflammatory transcription factors, including NFkB [23].

The NLR family pyrin domain containing 3 (NLRP3) inflammasome is a fundamental factor of the innate immune system that regulates caspase-1 activation and the release of pro-inflammatory cytokines interleukin (IL)-1β/IL-18 in response to microbial infection and cellular damage. The NLRP3 inflammasome may be activated by different stimuli, and numerous molecular and cellular contexts, including ionic flux, mitochondrial dysfunction, and the production of ROS [24]. Various inflammatory pathologies that determine the loss of tissue functionality are often caused by virulent agents (bacteria, viruses, etc.), which are often associated with systemic consequences. For example, *Porphyromonas gingivalis* (G), a gram-negative bacterium, is the main pathogen of periodontal diseases [25,26,27]. Periodontitis is an inflammatory condition caused by a condition of dysbiosis under the influence of environmental factors (smoking, pathogens, socioeconomic), systemic factors related to the host (diabetes, other inflammatory conditions, stress, anxiety) and genetic alterations. If left untreated, periodontitis leads to tissue damage which results in bone destruction and eventual tooth loss [28]. Based on the literature, in periodontal disease condition, histone acetylation induces the transcription of inflammatory genes such as p300/cAMP-regulated-enhancer (CRE)-binding protein (CREB)-binding (CBP) histone acetyltransferase, NFkB and other pro-inflammatory cytokines [29,30]. DNA methylation of cytosine residues is a crucial epigenetic modification that is fundamental for gene transcription and plays a critical part in inflammatory and immune responses [31]. DNMT1 modulates the methylation level of gene promoters, thus mediates the transcription of pro-inflammatory cytokines, including IL-6, IL-8 and tumor necrosis factor-α (TNF-α) [32]. DNA methylation could also affect inflammatory reactions by modulating the activation levels of crucial proteins of the NFkB and/or MAPK pathways. DNA methylation epigenetically modulates the transcription of TLRs and signal transduction molecules, including MyD88. This indicates that DNA methylation is involved in signaling pathways linked with inflammation [33].

The development of an in vitro model using hPDLSCs and endothelial-differentiated hPDLSCs (e-hPDLSCs) represents a good starting point to evaluate the intracellular mechanism activated during the inflammatory process caused by the LPS of *Porphyromonas gingivalis* (LPS-G). This research is focused on the effect of the bioactive component of Cur in the form of liposomal formulations (CurLIP) on the LPS-G dependent inflammatory process, in hPDLSCs and e-hPDLSCs with the aim to evaluate its anti-inflammatory properties.

## 2. Results

### 2.1. Human PDLSCs Showed the MSCs Profile

**Figure 1:**
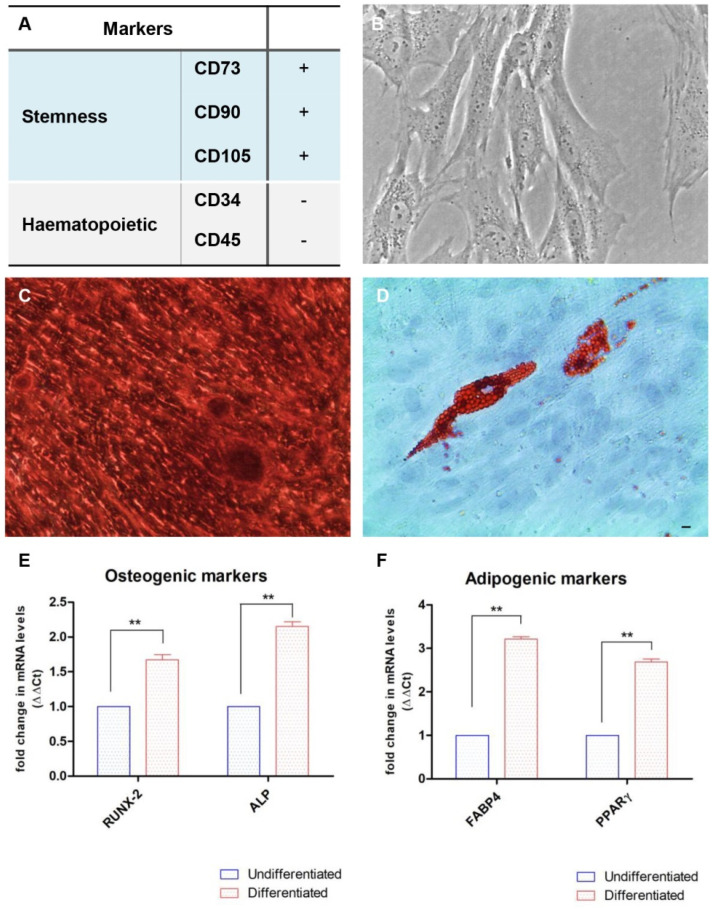
Characterization of human Periodontal Ligament Stem Cells (hPDLSCs). (**A**) Human PDLSCs were positive for CD73, CD90, and CD105, but negative for CD34 and CD45. +: positive expression; -: negative expression. (**B**) Cells were able to adhere on a plastic-substrate with a fibroblast-like morphology. (**C**,**D**) Human PDLSCs showed their potential to differentiate towards osteogenic (Alizarin Red S staining) and adipogenic (Oil Red O staining) lineages. (**E**,**F**) Gene expression of osteogenic and adipogenic-related markers. Scale bar: 20 µm. ** *p* < 0.01.

The table shown in Figure 1 demonstrated the positive expression of stemness markers CD73, CD90 and CD105, but not the expression of hematopoietic markers CD34 or CD45. The pluripotency ability of hPDLSCs was confirmed by the capacity to differentiate into adipogenic and osteogenic lineages in vitro. Cells were positive to specific in vitro staining, using alizarin red and adipo oil red solution. RT-PCR showed the positive expression of osteogenic and adipogenic-related markers, which further validated the cell differentiation ability (Figure 1).

### 2.2. Characterization of CurLIP

**Figure 2:**
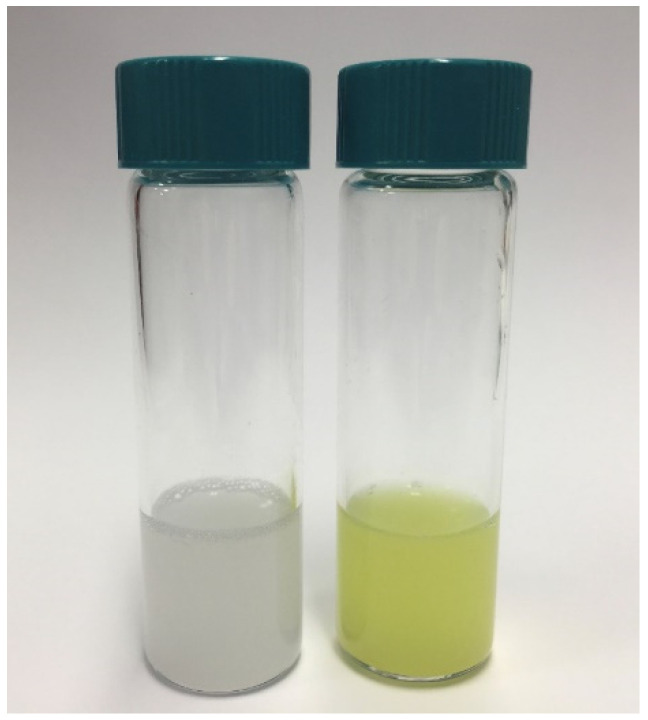
Liposomes loaded with curcumin (CurLIP) development. On the left photograph of liposomes before (left vial) and after (right vial) the addition of CUR. On the right table reporting dimensions (nm) and ζ-potential of liposomes in the absence and in the presence of CUR.

CurLIP were characterized by dynamic laser light scattering in terms of dimensions and ζ-potential. Both size and superficial charge varied on embedding Cur in the liposomes as expected for a lipophilic molecule that enters the bilayer by exposing its hydrophilic head group (the phenolic moiety) at the water-bilayer interface towards the aqueous environment (Figure 2).

### 2.3. Expression Levels of TLR4/MyD88/NFkB/NLRP3/Caspase-1/IL-1β in CurLIP and LPS-G Treated Cells

**Figure 3:**
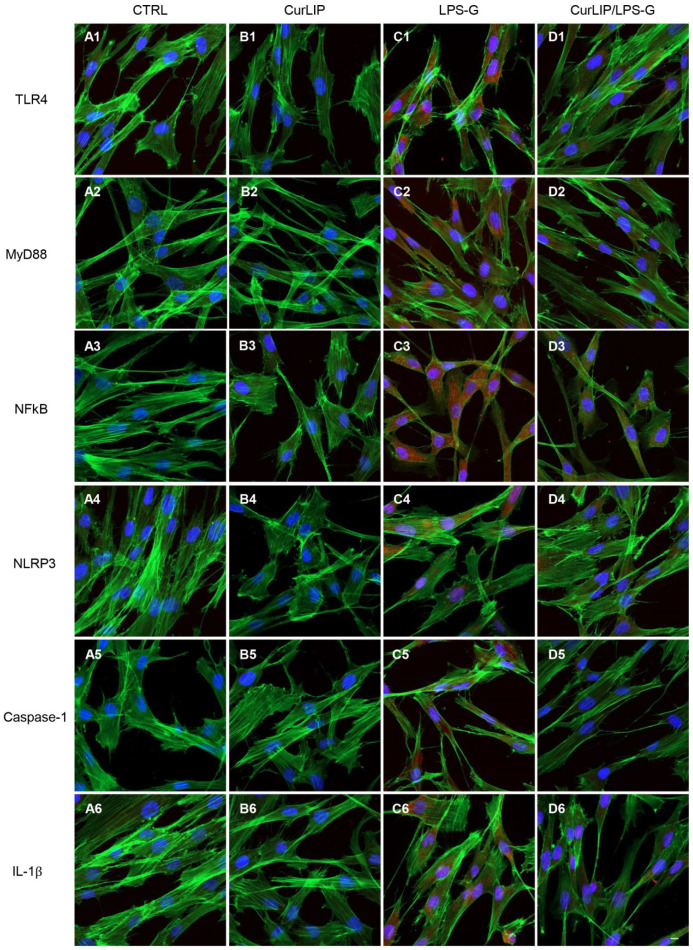
TLR4/MyD88/NFkB/NLRP3/Caspase-1/IL-1β protein expression in hPDLSCs. The localization of TLR4/MyD88/NFkB/NLRP3/Caspase-1/IL-1β was determined by immunofluorescence in all considered sample groups. Positive cells showed the protein expression in red, the cytoskeleton actin was marked in green and cell nuclei were stained with TOPRO in blue. (**A1**–**D1**) TLR4 expression in CTRL, CurLIP, LPS-G, CuRLIP/LPS-G. (**A2**–**D2**) MyD88 expression in CTRL, CurLIP, LPS-G, CuRLIP/LPS-G. (**A3**–**D3**) NFkB expression in CTRL, CurLIP, LPS-G, CuRLIP/LPS-G. (**A4**–**D4**) NLRP3 expression in CTRL, CurLIP, LPS-G, CuRLIP/LPS-G. (**A5**–**D5**) Caspase-1 expression in CTRL, CurLIP, LPS-G, CuRLIP/LPS-G. (**A6**–**D6**) IL-1β expression in CTRL, CurLIP, LPS-G, CuRLIP/LPS-G. Scale bar = 10 µm.

**Figure 4:**
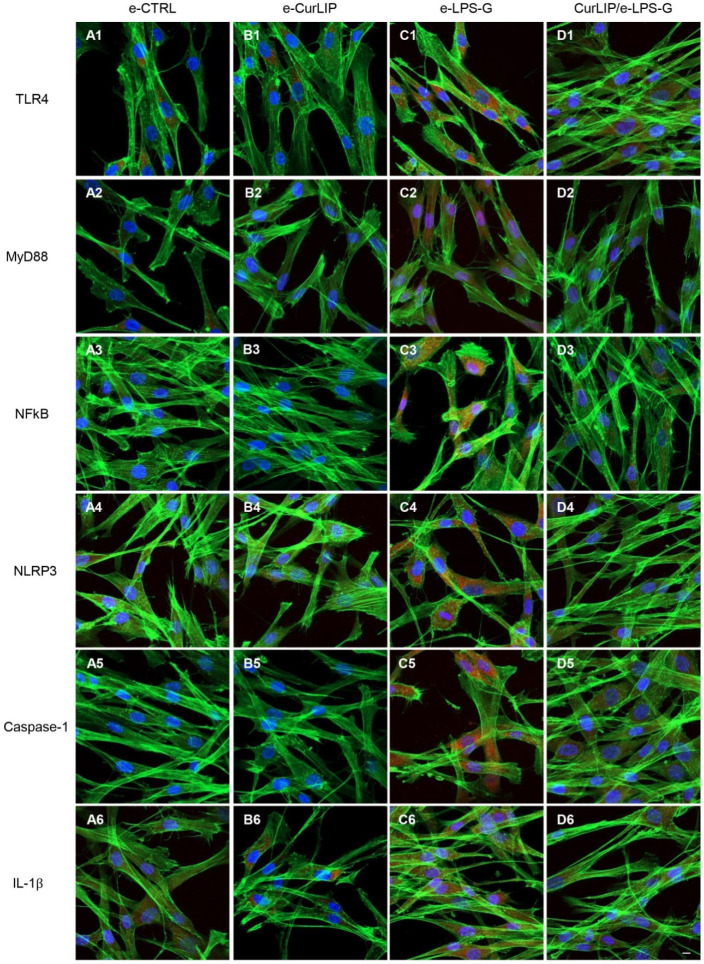
TLR4/MyD88/NFkB/NLRP3/Caspase-1/IL-1β protein expression in e-hPDLSCs. The localization of TLR4/MyD88/NFkB/NLRP3/Caspase-1/IL-1β was determined by immunofluorescence in all considered sample groups. Positive cells showed the protein expression in red, the cytoskeleton actin was marked in green and cell nuclei were stained with TOPRO in blue. (**A1**–**D1**) TLR4 expression in e-CTRL, e-CurLIP, e-LPS-G, CuRLIP/e-LPS-G. (**A2**–**D2**) MyD88 expression in e-CTRL, e-CurLIP, e-LPS-G, CuRLIP/e-LPS-G. (**A3**–**D3**) NFkB expression in e-CTRL, e-CurLIP, e-LPS-G, CuRLIP/e-LPS-G. (**A4**–**D4**) NLRP3 expression in e-CTRL, e-CurLIP, e-LPS-G, CuRLIP/e-LPS-G. (**A5**–**D5**) Caspase-1 expression in e-CTRL, e-CurLIP, e-LPS-G, CuRLIP/e-LPS-G. (**A6**–**D6**) IL-1β expression in e-CTRL, e-CurLIP, e-LPS-G, CuRLIP/e-LPS-G. Scale bar = 10 µm.

Immunofluorescence results showed that TLR4/MyD88/NFkB/NLRP3/Caspase-1/IL-1β were expressed in hPDLSCs treated with LPS-G for 24 h compared to the untreated cells. Cells co-treated with CurLIP and LPS-G showed a drastic reduction of inflammatory protein levels. The CurLIP treatment alone does not affect the protein expression and remains similar to the untreated hPDLSCs (Figure 3). In e-hPDLSCs the LPS-G treatment showed the same inflammatory pathway, indeed, treated cells demonstrated a high level of TLR4/MyD88/NFkB/NLRP3/Caspase-1/IL-1β when compared to the untreated samples. The CurLIP treatment showed a decrease of the expression of inflammation proteins (Figure 4).

### 2.4. LPS-G Induced a Down Expression of DNMT1 and an Over Expression of p300

**Figure 5:**
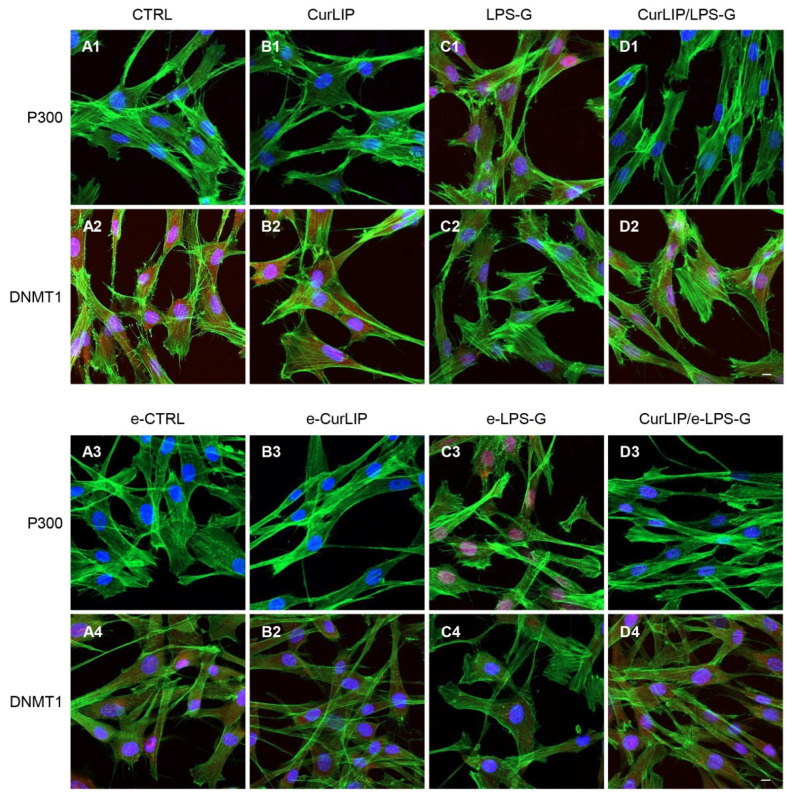
DNMT1 and p300 expression in hPDLSCs and e-hPDLSCs. The localization of DNMT1 and p300 was determined by immunofluorescence in all considered sample groups. Positive cells showed the protein expression in red, the cytoskeleton actin was marked in green and cell nuclei were stained with TOPRO in blue. (**A1**–**D1**) p300 expression in CTRL, CurLIP, LPS-G and CurLIP/LPS-G. (**A2**–**D2**) DNMT1 expression in CTRL, CurLIP, LPS-G and CurLIP/LPS-G. (**A3**–**D3**) p300 expression in e-CTRL, e-CurLIP, e-LPS-G and CurLIP/e-LPS-G. (**A4**–**D4**) DNMT1 expression in e-CTRL, e-CurLIP, e-LPS-G and CurLIP/e-LPS-G. Scale bar = 10 µm.

**Figure 6:**
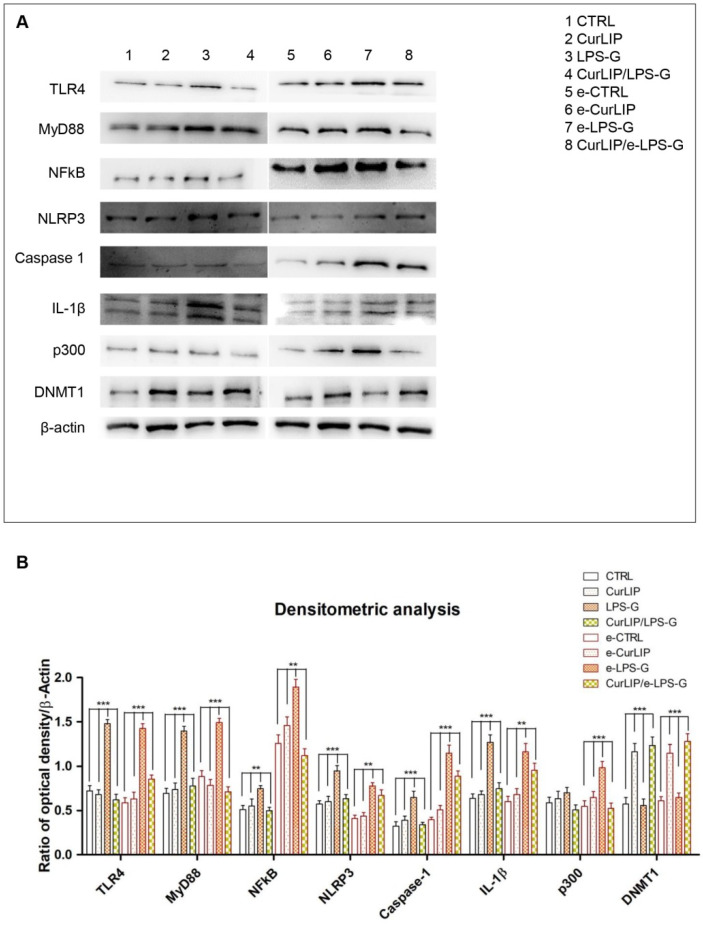
TLR4, MyD88, NFkB, NLRP3, Caspase-1, IL-1β, DNMT1, and p300 protein quantization in hPDLSCs. (**A**) Protein levels of TLR4, MyD88, NFkB, NLRP3, Caspase-1, IL-1βDNMT1 and p300 were determined by Western blotting using specific antibodies. (**B**) Relative ratio of TLR4, MyD88, NFkB, NLRP3, Caspase-1, IL-1β, DNMT1 and p300 normalized with β-actin. *** *p* < 0.001; ** *p* < 0.01.

DNMT1 was negatively regulated in hPDLSCs and e-hPDLSCs exposed to LPS-G inflammatory stimulus, while control untreated hPDLSCS and e-hPDLSCs showed no change in the basal expression. Interestingly, the expression of DNMT1 was similar to the basal cells in hPDLSCs and e-hPDLSCs treated with CurLIP and the cells that underwent co-treatment (CurLIP/LPS-G) showed a gradual increase in DNMT1 level (Figure 5). The expression of p300 was positively regulated when cells were exposed to LPS-G, while the expression was restored when hPDLSCs and e-hPDLSCs were co-treated with CurLIP/LPS-G (Figure 5A1–D1). These results indicate that in vitro co-treatment by CurLIP/LPS-G may induce a restoration of physiological expression of DNMT1 and p300 in hPDLSCs and e-hPDLSCs (Figure 5). All confocal microscopy images were confirmed by Western blot analyses in order to demonstrate the quantization of protein level (Figure 6).

### 2.5. CurLIP Treatment Attenuates ROS Production in LPS-G Treated Cells

**Figure 7:**
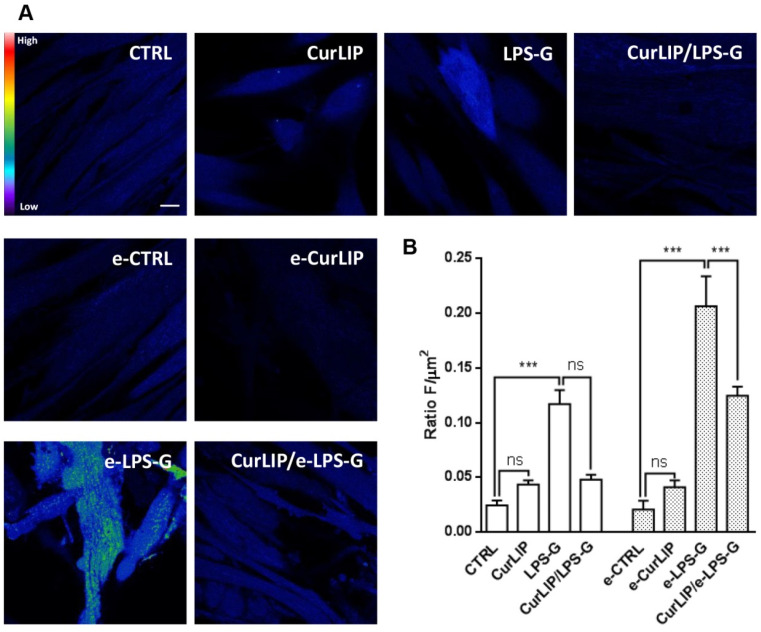
ROS measurements. (**A**) Representative images of H2DCFDA loaded cells pseudocolored from black to red indicating low/absent to high levels of ROS. (**B**) Analysis of ROS production calculated as arbitrary unit of fluorescence per cell surface unit (F/µm^2^). Data are expressed as mean± S.E.M (CTRL *n* = 166, CurLiP *n* = 130, LPS-G *n* = 117, CurLiP/LPS-G *n* = 114; e-CTRL = 85, e-CurLiP = 121, e-LPS-G *n* = 197, e-CurLiP/LPS-G *n* = 155, N = 3, *** *p* < 0.001). Statistical analysis was performed by One-way ANOVA and post hoc Bonferroni Scale Bar = 10 µm.

ROS production induced by LPS-G has been studied in hPDLSCs and e-hPDLSCs loaded with the cell-permeant ROS probes H2DCFDA. Once diffused inside the cell, the reaction with intracellular esterases switched the molecule into the active form. In this way the nonfluorescent H2DCFDA is converted to the highly fluorescent 2′,7′-dichlorofluorescein (DCF) by ROS. Images were acquired in live cells by means of confocal microscopy as reported in Figure 7A and the single cells fluorescence recorded was off-line analyzed. Quantitative results (Figure 7B) showed a significant increase in ROS production both in 5 µg mL^−1^ LPS-G treated- hPDLSCs and e-hPDLSCs (LPS-G and e-LPS-G respectively) vs the relative control condition (CTRL for hPDLSCs and e-CTRL e-hPDLSCs, respectively). Comparing the response between hPDLSCs and e-hPDLSCs at 5 µg mL^−1^ LPS-G it is observed that these latter ones appeared more prone to produce ROS. Indeed, for undifferentiated hPDLSCs we found a mean value about half with respect to the endothelial-differentiated one (0.12 ± 0.01 vs 0.21 ± 0.03, mean ± SEM *** *p* < 0.001). Interestingly, the co-treatment of LPS-G together with CurLIP counteracted the LPS-G increase in ROS production (0.21 ± 0.03, vs 0.12 ± 0.01, mean ± SEM *** *p* < 0.001), while in the undifferentiated cells, the reduction, even if present, appeared to be not statistically significant.

## 3. Discussion

To date, several researchers are exploring the biological response of MSCs treated with antioxidant molecules to the inflammatory events.

The minimal criteria for defining human MSCs were established by Dominici et al., in 2006. The cell population to be identified as MSC must show the ability to adhere to a plastic surface, a fibroblast-like morphology, the expression of stemness markers as CD105, CD73 and CD90, and ultimately, the lack of expression of hematopoietic markers such as CD14, CD34 and CD45 [34]. MSCs are recognized as valuable cell source for tissue renewal, exhibiting a critical role in tissue engineering and regenerative medicine field [35]. Many scientists focus their attention on MSCs isolated from the human oral cavity due to their easy access, multi-lineage differentiation abilities and high proliferation capability [36]. In particular, oral derived-MSCs such as hPDLSCs are capable of controlling the homeostasis of tooth and contribute to tissue regeneration [37]. They also evidenced the capacity of MSCs to defend against infectious agents due to their immunomodulatory properties [38].

Due to the complexity and the limitations still present in the periodontal disease treatments, it is urgently necessary to develop multi-targeted, cost-effective, non-toxic, and highly potent molecule for the management of this multifactorial disease.

Cur, a yellow pigment, is the principal active factor of Indian spice Turmeric (Curcuma longa), which was isolated for the first time two centuries ago in 1815 by two German Scientists, Vogel and Pelletier.

The Curcuma longa, a largely cultivated tropical plant, has been utilized since ancient times as a spice, as a beauty care agent, and as a traditional medicine [39].

Curcumin is a bioactive polyphenolic molecule recognized in turmeric, which has been collectively denoted as curcuminoids [40]. In the last decades, Cur has gained a lot of attention in the scientific field due to its outstanding properties such as antibacterial, anti-inflammatory, hypolipidemic, hepatoprotective, anti-cancer, anti-diabetic, anti-aging as reported by previous findings obtained from in vitro and in vivo studies and clinical trials [41]. This multitargeted molecule has been demonstrated to show anti-inflammatory action through the reduction of several intracellular mechanism including NF-κB, STAT3, Nrf2, ROS, and COX-2 at a molecular level [42].

Furthermore, the safety, tolerability, and nontoxicity of Cur has been largely recognized by human clinical trials [43]. Clinical trials demonstrated that Cur at 8 g/day was safe and well-tolerated without showing any side effects. The pharmacokinetics of liposomal gel loaded with Cur exhibited that nano-sized liposomes are capable to enter in 1 h in both strata of corneum and skin [44,45].

Based on these important notions, the present work aimed at developing an in vitro model for evaluating the potential positive effect of CurLIP utilized at 20 μM in re-establishing the homeostasis in hPDLSCs after LPS-G inflammatory exposure.

The prepared CurLip demonstrated a small increase of dimension and a decrease of ζ-potential when Cur is inserted in the bilayer, similarly to previously published evidence of inclusion of Cur in the bilayer [Curcumin/Liposome Nanotechnology as Delivery Platform for Anti-inflammatory Activities via NFkB/ERK/pERK Pathway in Human Dental Pulp Treated With 2-HydroxyEthyl MethAcrylate (HEMA)] and partial deprotonation of phenolic moieties (pKa = 8.11 ± 0.46, calculated using Advanced Chemistry Development (ACD/Labs) at the buffered pH.

*Porphyromonas gingivalis*, a Gram-negative anaerobic bacterium, is one of the prime etiological agent involved in the pathogenesis and advancement of the inflammatory events of periodontal disease [46]. LPS, the major constituent of outer membrane of *Porphyromonas gingivalis*, is retained to be a responsible factor of the periodontitis virulence.

Periodontal disease is characterized by an inflammatory pathologic state of the gingiva and the supporting structures of the periodontium, which enclose gingival, alveolar bone, periodontal ligament, and cementum. The disease starts as acute inflammation of the gingival tissue, and untreated infections can bring to teeth pockets formations, and ultimately teeth destruction [47]. The periodontal chronic inflammation could be related to the CVD, several studies demonstrated an association between periodontitis and CVD, and there is increasing evidence that periodontal disease can have negative cardiovascular effects [48].

In the present study, the experiments were performed in order to evaluate the effect of CurLIP formulation against the inflammation molecular cascade sustained by LPS-G. Human PDLSCs were stimulated using LPS-G to mimic the periodontal microenviroment in their vitro condition. Moreover, the use of e-hPDLSCs was essential to evaluate the intracellular signaling pathway that may link the periodontal and CVD.

LPS-G plays a fundamental part in contributing inflammation and promoting cells to release pro-inflammatory cytokines such as IL-1β, TNF-α and IL-6. Moreover, LPSs are well-known to induce the production of pro-inflammatory cytokines, mostly through TLR 4 and NFkB [49].

NLRP3 is the most extensively studied inflammasome and has been linked with several disorders characterized by chronic inflammation, which includes cancer, type 2 diabetes and rheumatoid arthritis, atherosclerosis and periodontal diseases [50]

Several stimuli and various molecular and cellular events, enclosing ionic flux, mitochondrial failure, and the release of ROS has been demonstrated to trigger the activation of NLPR3 [51].

NLRP3 inflammasome activation is divided into the priming and activation phases. The priming step is ensured by inflammatory stimuli such as TLR4 agonists, which induce NFkB-mediated NLRP3 and pro-IL-1β expression, and the activation step is triggered by pathogen-associated molecular patterns (PAMPs) and damage-associated molecular patterns (DAMPs), thus inducing NLRP3 inflammasome assembly and caspase-1-mediated IL-1β and IL-18 secretion [52].

As evidenced by immunofluorescence and western blotting analyses, the hPDLSCs stimulated with LPS-G showed an upregulation of TLR-4, MyD88, NFkB, NLRP3, Caspase-1 and IL-1β expression level, while the cells treated with the CurLIP in combination with LPS-G exhibited a remarkable reduction of the inflammatory molecules, suggesting a re-establishment of conditions reported in untreated hPDLSCs [53]. It is well known that MyD88, a fundamental adaptor protein for most TLRs, mediates the stimulation of inflammatory cytokines through NFkB [54,55]. Furthermore, in the periodontal disease, histone acetylation promotes the transcription of inflammatory genes such as p300/CBP histone acetyltransferase, NFkB and other pro-inflammatory cytokines. On this basis, we investigated the p300/DNMT1 level expression in the hPDLSCs and in e-hPDLSCs treated with LPS-G, or CurLIP and/or co-treated with CurLIP/LPS-G.

Our results reported a significant high level expression of p300 when both hPDLSCs and e-hPDLSCs were treated with LPS-G, while the cells co-treated with CurLIP/LPS-G revealed a similar level of expression of p300 reported in untreated cells, hypothesizing beneficial effects due to CurLIP treatment. In parallel, DNMT1 level expression was also evaluated through immunofluorescence and protein analyses, evidencing a lower expression in cells treated with LPS-G and higher expression in CurLIP alone or in combination with LPS-G. These results suggested that LPS-G may activate the inflammatory cascade and the p300 signal transduction induces the NFkB nuclear translocation and the downregulation of DNMT1, as previously reported [30,56]. In the same in vitro model, ROS production was also investigated [57].

Our data evidenced high level of ROS production in LPS-G treated cells. Interestingly, endothelial-differentiated hPDLSCs appear to be more susceptible, producing more ROS than undifferentiated counterparts. When produced at physiological levels, ROS act as crucial second messengers which transduce intracellular signals in biological processes. On the other hand, when an anomalous production of ROS exceeds the buffering capacity of the antioxidant defense system an oxidative stress occurs. In our model the supply of Cur in LPS-G-treated cells were able to reduce the ROS only in endothelial-differentiated hPDLSCs. This could be due to different metabolic features induced by differentiation as reported for endothelial cells and endothelial progenitor cells in terms of ROS production and glutathione peroxidase type 1 expression and activity [58]. Moreover, our results are in accordance with the results found by Yong Sook and coll., were in human umbilical vein endothelial cells, Cur at concentration of 10–30µM reduced, but not abolished the ROS production induced by TNF-α and attenuated the inflammatory response [59].

## 4. Materials and Methods

### 4.1. Human Periodontal Ligament Stem Cells (hPDLSCs) Isolation and Culture Establishment

Written approval for human periodontal ligament biopsy collection was obtained from the Medical Ethics Committee at the Medical School, “G. d’Annunzio” University, Italy (number 266/17 April 2014), and each participant viewed, completed, and signed the informed consent. Human PDLSCs were collected from healthy human periodontal ligament tissue of human premolar teeth removed before starting the orthodontic treatment. In the study three patients have been enrolled, all donors were free from oral and systemic diseases with a good health condition [60]. After extraction, the periodontal ligament fragments were crumbled and washed five times with phosphate buffered saline (PBS, Lonza, Basel, Switzerland). At this point, fragments tissue was placed in MSCBM-CD (Lonza) and then incubated in a humidified atmosphere with 5% CO_2_ at 37 °C, replacing medium every two days to stimulate the growth of human mesenchymal stem cells [61]. It has been reported that after two weeks the cells migrated spontaneously from the explants. Cells were maintained in MSCBM-CD (Lonza), and those in passage 2 were used for this experiment plating at 1 × 10^3^ cells/cm^2^density.

### 4.2. FACS Analysis

Human PDLSCs at the passage 2 were treated with 0.1% trypsin-EDTA, harvested and suspended in PBS and stained with the following markers: Fluorescein isothiocyanate-conjugated anti-CD45 (CD45 FITC) and FITC-conjugated anti-CD105 (CD105 FITC) were obtained from Ancell (MN, USA); Phycoerythrin-conjugated anti-CD73 (CD73 PE), FITC-conjugated anti-CD90 (CD90 FITC), and PE-conjugated anti-CD34 (CD34-PE) were purchased from Beckman Coulter (Fullerton, CA, USA). 5 × 10^5^ cells were incubated with 100 mL of 20 mM ethylenediamintetraacetic acid (EDTA) at 37 °C for 10 min. Cells were washed with 3 mL of washing buffer and centrifuged (4 °C, 400× *g*, 8 min). For antigens detection, samples were resuspended in 100 mL washing buffer containing the appropriate amount of surface antibodies, incubated for 30 min at 4 °C in the dark, washed (3 mL of washing buffer), centrifuged (4 °C, 400× *g*, 8 min), resuspended with 1 mL 0.5% paraformaldehyde, incubated for 5 min at room temperature, washed, centrifuged (4 °C, 400× *g*, 8 min) and stored at 4 °C in the dark until acquisition. Cells were analyzed using a FACSCalibur flow cytometer (Becton-Dickinson, Mountain View, CA, USA), using CellQuestTM software (Becton-Dickinson). Quality control included regular check-ups with Rainbow Calibration Particles (Becton-Dickinson). Debris was excluded from the analysis by gating on morphological parameters; 20,000 non-debris events in the morphological gate were recorded for each sample. To assess non-specific fluorescence, we used isotype controls. All antibodies were titrated under assay conditions and optimal photomultiplier voltages were established for each channel. Data were analyzed using FlowJoTM software (Becton-Dickinson,) [62].

### 4.3. Analysis of Mesengenic Differentiation of hPDLSCs with Colorimetric Detection and RT-PCR

Since the hPDLSCs are capable of differentiating into mesengenic lines, a colorimetric detection was used to confirm the stemness of the cells considered, which was then corroborated by a reverse transcription polymerase chain reaction (RT-PCR) analysis. Starting from the colorimetric analysis, to identify the osteogenic differentiation Alizarin red S solution (Sigma-Aldrich, Milan, Italy) was used, which highlighted calcium deposits. For this experiment, hPDLSCs were cultured in a 24-well multiwell with a density of 2 × 10^4^ cells/well, using MSCBM-CD supplemented with 10 nmol/L dexamethasone, 10 nmol/L beta glycerophosphate (Sigma-Aldrich), and 50 mmol/L ascorbic acid for 21 days. For the adipogenic differentiation hPDLSCs were grown in a 24-well multiwell with a density of 2 × 10^4^ cells/well, in MSCBM-CD supplemented with 10 mmol/L dexamethasone, 10 nmol/L 3-isobutyl-1-methylxanthine, 5 mg/mL insulin and 60 mmol/L indomethacin for 28 days. Medium was changed for every 3 days. Oil Red O solution (Sigma-Aldrich) stains the lipid droplet at the cytoplasmic level, allowing to identify the adipogenic phenotype. Leica DMIL inverted light microscope can visualize the occurred hPDLSCs differentiation. To confirm the results of colorimetric detection by RT-PCR, the expression of specific genes of osteogenic and adipogenic differentiation were analyzed. In particular, the following factors were monitored: the transcription factor related to Runt-2 (RUNX-2) and alkaline phosphatase (ALP), after 7 days in osteogenic differentiated culture, the fatty acid-binding protein 4 (FABP4) and the peroxisomal proliferator-activated γ receptor (PPARγ), after 28 days of differentiated adipogenic culture. Commercially available TaqMan Gene Expression Assays (RUNX-2 Hs00231692_m1; ALP Hs01029144_m1; FABP4Hs01086177_m1; PPARγ Hs01115513_m1) and the Taq-Man Universal PCR Master Mix (Applied Biosystems, Foster City, CA, USA) were used according to standard protocols. Beta-2 microglobulin (B2M Hs99999907_m1) (Applied Biosystems) was used for template normalization. Real-time PCR was performed in three independent experiments, and duplicate determinations were carried out for each sample [63].

### 4.4. Endothelial Differentiation

To induce the endothelial commitment, the hPDLSCs were maintained in differentiation medium for 14 days as previously described [64]. Cells were cultured in Endothelial growth medium (EGM-2, Lonza), supplemented with hydrocortisone, human Fibroblast Growth Factor (hFGF-b), R3-Insulin-like Growth Factor-1 (R3-IGF-1), ascorbic acid, human Epithelial Growth Factor (hEGF), GA-1000, heparin, 5% FBS and 50 ng/mL of Vascular Endothelial Growth Factor-165 (VEGF-165).

### 4.5. Liposomes loaded with Curcumin (CurLIP)

Liposomes were prepared by thin film hydration method. A proper amount of POPC (1-palmitoyl-2-oleoyl-phosphatidylcholine, Avanti Polar Lipids, Alabama, United States), dissolved in chloroform, was added to a round-bottomed flask and dried using a rotary evaporator under reduced pressure at 40 °C until a thin opaque film was observed on the bottom of the flask. The phospholipid film was further dried under vacuum and kept at 4 °C overnight. Hydration of the lipid film was carried out by adding PBS buffer (pH 7.4) at room temperature with constant agitation for 30 min. The obtained multivesicular suspension was sonicated for 30 min and then subjected to sterilization under UV lamp for 2 h [65,66]. An appropriate amount of Cur in DMSO was added to the liposomal suspension to obtain a POPC to Cur molar ratio of 25:1. For the in vitro test POPC and Cur concentrations in the liposomal suspension were 10 mM and 0.4 mM, respectively. 100 µL of this liposomal suspension were added to a final volume of medium of 2 mL in order to obtain Cur concentration of 20 µM. The size and ζ-potential of pure and curcumin loaded POPC liposomes were determined in diluted samples using a 90Plus/BI-MAS ZetaPlus multiangle size analyzer (Brookhaven Instrument Corp., Holtsville, NY, USA) (Figure 2).

### 4.6. Study Design

All experiments were performed in triplicate with hPDLSCs at passage 2.

The study was organized into the following groups:

- Untreated hPDLSCs, used as the negative control (CTRL)
- hPDLSCs treated for 24 h with 10 mmol L^−1^ with Liposome enriched with Curcumin (CurLIP)
- hPDLSCs treated for 24 h with ultrapure lipopolysaccharide from *P. gingivalis* (InvivoGen, San Diego, CA, USA) 5 μg mL^−1^ (LPS-G)
- hPDLSCs co-treated for 24 h with 10 mmol L^−1^ CurLIP and LPS-G 5 μg mL^−1^ (CurLIP/LPS-G)
- Untreated e-hPDLSCs, used as the negative control (e-CTRL)
- e-hPDLSCs treated for 24 h with 10 mmol L^−1^ CurLIP(e-CurLIP)
- e-hPDLSCs treated for 24 h with 5 μg mL^−1^ LPS-G (e-LPS-G)
- e-hPDLSCs co-treated for 24 h with 10 mmol L^−1^ CurLIPand 5 μg mL^−1^ LPS-G (CurLIP/e-LPS-G)

### 4.7. Immunofluorescence Analysis and Confocal Laser Scanning Microscope (CLSM)

In the present work, the cells were subjected to immunofluorescence and CLSM analysis for the expression of TLR4, MyD88, NFkB, NLRP3, Caspase-1, IL-1β, p300 and DNMT1. For this purpose, samples were first fixed with a 4% solution of paraformaldehyde in 0.1 M PBS (Lonza), then the cells were permeabilized by treating them with 0.5% Triton X-100 in PBS for 10 min. Saturation was then performed with 5% skim milk in PBS for 30 min. Later, the incubation was carried out for 1 h at room temperature, with the primary antibodies specific for the markers considered, diluted in 2.5% of milk: anti-TLR4 (1:200, Santa Cruz Biotechnology, Santa Cruz, CA, USA), anti-MyD88 (1:500, Santa Cruz Biotechnology), anti-NFkB p65 (1:500, Santa Cruz Biotechnology), anti-NLRP3 (3 μg/mL, Novus, MIaln, Italy), anti-Caspase-1 (1:500, Santa Cruz Biotechnology), anti-IL-1β (1 μg/mL, Termofisher, Milan, Italy); anti-p300 (10 µg/mL, OriGene Technologies, Rockville, MD, USA) and anti-DNMT1 (1:100, OriGene). At the end of the incubation with the primary antibodies, the incubation was carried out for 1 h at 37 ° C with the secondary antibodies capable of highlighting the nuclei (TOPRO 1:200, Molecular Probes) and the cytoskeletal actin (green fluorescent conjugate of the falloidin Alexa Fluor 488 1: 200, Molecular Probes) [41,42]. The Zeiss LSM800META confocal system (Zeiss, Jena, Germany) with a Plan Neofluar oil immersion objective (63×) was used to visualize the stained cells with the described procedure. The excitation lines at 488 nm for the argon laser beam and at 543 and 665 nm for a helium-neon source made it possible to obtain the different micrographs.

### 4.8. Western Blot Analysis

Western blot analysis was performed as previously described by Gugliandolo et al. [67]. The primary antibodies used in the procedure were: TLR4 (1:500, Santa Cruz Biotechnology), MyD88 (1:500, Santa Cruz Biotechnology), NFkB (1:500, Santa Cruz Biotechnology), NLRP3 (3 μg/mL, Novus), caspase-1 (1:500, Santa Cruz Biotechnology), IL-1β (1 μg/mL, Termofisher), p300 (1:750, OriGene) and anti-DNMT1 (1:750, Origene); β-actin (1:750, Santa Cruz Biotechnology) was used as a normalizer to ensure loading uniformity. Lastly, the ECL method by means of Alliance 2.7 (UVItec Limited, Cambridge, UK) was used for the identification and quantification of the obtained bands.

### 4.9. ROS Evaluation

Undifferentiated- and endothelial-differentiated hPDLSCs were seeded in 35 mm imaging dish (µ-Dish, ibidi GmbH, Gräfelfing, D) and treated for 24 h in culture medium containing 5 µg mL-1 LPS-G; or 5 µg mL-1 LPS-G plus 10 mmol L^−1^ Liposome enriched with Curcumin or 10 mmol L^−1^ Liposome enriched with Curcumin alone or culture medium alone. At the end of expected time, incubation media was removed and the cells were washed with Normal External Solution (NES) containing (in mM): 125 NaCl, 5 KCl, 1 MgSO_4_, 1 KH_2_PO_4_, 5.5 glucose, 1 CaCl_2_, 20 HEPES, pH 7.4 and incubated with 10 μM of 2′,7′-dichlorodihydrofluorescein diacetate (H2DCFDA, Thermo Fisher Scientific) in NES at 37 °C in humidified incubator (for 30 min). During the probes’ incubation, they were kept constant all the respective culture media treatments. At the end of dye incubation, the cells were washed with NES and observed in NES alone (CTRL, or e-CTRL) or NES plus LPS-G, LPS-G, and CurLIP or CurLIP. For each conditions, confocal images were randomly acquired by means of motorized table SMC 2009 and multiple single position acquisition function (tiles mode, advanced setup) of Zen Blue software using a Zeiss LSM800 microscope (Carl Zeiss, Jena, Germany), equipped with an inverted microscope Axio-obserber. D1 and an objective W-Plan-Apo 40X/1.3 DIC. Excitation was fixed at 488 nm and emission collected setting the filter set over 505–530 nm. The acquisition settings were maintained constant between specimens. Off-line image analyses were performed using Fiji distribution of ImageJ measuring for each acquired cell the mean of fluorescence intensity (arbitrary units, F) and the area of the measured cells (µm^2^) [68]. Quantitative data of ROS production is expressed as ratio F/µm^2^.

### 4.10. Statistical Analysis

Statistical evaluation was performed with *t*-test and ordinary one-way ANOVA followed by post hoc Bonferroni′s multiple comparisons tests using GraphPad Prism 4.0 software (Graph-Pad, San Diego, CA, USA). Values of *p* < 0.01 were considered statistically significant.

## 5. Conclusions

Taken together, these results demonstrated that CurLIP formulation is able to decrease the inflammatory effects triggered by LPS-G in hPDLSCs and e-hPDLSCs, highlighting the promising role played by CurLIP as an anti-inflammatory potential treatment in periodontal disorder. These findings suggest that CurLIP formulation may represent a novel strategy for re-establishing the tissue homeostasis through the modulation of TLR4/MyD88/NFkB/NLRP3/Caspase-1/IL-1β signaling cascade in an in vitro model of hPDLSCs and e-hPDLSCs exposed to LPS-G stimulus and may be considered as a promising molecule to be used alone or in combination therapy for the periodontal disorder. Further studies are needed to determine the role of these pathways in periodontitis and in cardiovascular disease and to evaluate the mechanisms involved in the regulatory processes.

## Data Availability

Data are available upon request.
